# Daily Exposure to Air Pollution Particulate Matter Is Associated with Atrial Fibrillation in High-Risk Patients

**DOI:** 10.3390/ijerph17176017

**Published:** 2020-08-19

**Authors:** Elisa Gallo, Franco Folino, Gianfranco Buja, Gabriele Zanotto, Daniele Bottigliengo, Rosanna Comoretto, Elena Marras, Giuseppe Allocca, Diego Vaccari, Gianni Gasparini, Emanuele Bertaglia, Franco Zoppo, Vittorio Calzolari, Rene Nangah Suh, Barbara Ignatiuk, Corrado Lanera, Alessandro Benassi, Dario Gregori, Sabino Iliceto

**Affiliations:** 1 Unit of Biostatistics, Epidemiology and Public Health, Department of Cardiac, Thoracic, Vascular Sciences and Public Health, University of Padua, 35131 Padua, Italy; elisa.gallo@unipd.it (E.G.); daniele.bottigliengo@phd.unipd.it (D.B.); rosanna.comoretto@unipd.it (R.C.); corrado.lanera@unipd.it (C.L.); 2 Division of Cardiology, Department of Cardiac, Thoracic, Vascular Sciences and Public Health, University of Padua, 35128 Padua, Italy; franco.folino@unipd.it (F.F.); gianfranco.buja@unipd.it (G.B.); bertagliaferro@alice.it (E.B.); sabino.iliceto@unipd.it (S.I.); 3 Department of Cardiology, Mater Salutis Hospital, 37045 Legnago, Italy; gabzanot@tin.it; 4 Department of Cardiology, S Maria dei Battuti Hospital, 31015 Conegliano, Italy; elena.marras@alice.it (E.M.); alloccag75@libero.it (G.A.); 5 Department of Cardiology, Civic Hospital, 31044 Montebelluna, Italy; vaccaridiego@gmail.com; 6 Department of Cardiology, Dell’Angelo Hospital, 30174 Mestre, Italy; g_gasparini2000@yahoo.it; 7 Department of Cardiology, Civic Hospital, 30035 Mirano, Italy; francozoppo@gmail.com; 8 Department of Cardiology, Ca’ Foncello Hospital, 31100 Treviso, Italy; toiocalzolari@gmail.com; 9 Department of Cardiology, Civic Hospital, 30026 Portogruaro, Italy; renenangah@yahoo.com; 10 Department of Cardiology, Civic Hospital, 35043 Monselice, Italy; barbara.ignatiuk@fastwebnet.it; 11 Regional Agency for Environmental Prevention and Protection of the Veneto Region, 35121 Padua, Italy; alessandro.benassi@regione.veneto.it

**Keywords:** atrial fibrillation, air pollution, cardiovascular risk

## Abstract

Several epidemiological studies found an association between acute exposure to fine particulate matter of less than 2.5 μm and 10 μm in aerodynamic diameter (PM_2.5_ and PM_10_) and cardiovascular diseases, ventricular fibrillation incidence and mortality. The effects of pollution on atrial fibrillation (AF) beyond the first several hours of exposure remain controversial. A total of 145 patients with implantable cardioverter-defibrillators (ICDs), cardiac resynchronization therapy defibrillators (ICD-CRT), or pacemakers were enrolled in this multicentric prospective study. Daily levels of PM_2.5_ and PM_10_ were collected from monitoring stations within 20 km of the patient’s residence. A Firth Logistic Regression model was used to evaluate the association between AF and daily exposure to PM_2.5_ and PM_10_. Exposure levels to PM_2.5_ and PM_10_ were moderate, being above the World Health Organization (WHO) PM_2.5_ and PM_10_ thresholds of 25 μg/m^3^ and 50 μg/m^3^, respectively, on 26% and 18% of the follow-up days. An association was found between daily levels of PM_2.5_ and PM_10_ and AF (95% confidence intervals (CIs) of 1.34–2.40 and 1.44–4.28, respectively) for an increase of 50 µg/m^3^ above the WHO threshold. Daily exposure to moderate PM_2.5_ and PM_10_ levels is associated with AF in patients who are not prone to AF.

## 1. Introduction

The effects of air pollution on overall mortality and on cardiovascular mortality have been well documented [1,2]. Specifically, particulate matter has been linked to an increased risk of myocardial infarction [3] and ventricular fibrillation [4], whereas its relationship with atrial fibrillation (AF) has not been completely clarified yet. AF is the most common arrhythmia, affecting 4.5 million people in Europe [5], and its prevalence is projected to increase in the next years [6]. Moreover, AF is associated with an increased risk of hospitalization and stroke [7,8].

In the last two decades, some epidemiological studies evaluated the association between short-term exposure to air pollution and AF with two main approaches: analyzing hospital admissions [9,10,11,12] or enrolling subjects for whom continuous monitoring of the heart rhythm was possible (i.e., patients with implantable devices or subjects that underwent a Holter screening) [13,14,15]. Evidence of an association between AF onset and an increase in particulate matter of less than 2.5 μm in aerodynamic diameter (PM_2.5_) concentration in the 2 h prior to AF has been demonstrated by Link and colleagues [13] and for the whole day by Liu and colleagues [16]. Moreover, in a healthy community-dwelling sample of nonsmokers, AF predictors were found to be associated with increased levels of PM_2.5_ [17], and lagged alterations in the ECG were found in association with PM_2.5_ in patients undergoing catheterization [15].

Contrariwise, controlled exposure studies in both healthy volunteers and patients with coronary heart disease have not shown any pro-arrhythmic effect of air pollution [18], whereas a positive but not statistically significant association was found between PM_2.5_ exposure and hospitalization for both AF [19] and paroxysmal atrial fibrillation episodes [20]. Moreover, some studies on hospitalization rates for AF found an association with PM [9,10,16], whereas others highlighted a lack of association [11,12].

The present study aimed to enhance the knowledge about the potential association between PM exposure and AF in patients with implanted devices, such as cardiac resynchronization therapy defibrillators (ICD-CRT), implantable cardioverter defibrillators (ICD), or pacemakers, that allow for the detection of AF episodes.

## 2. Materials and Methods 

### 2.1. Patient Sample

Subjects were selected from the ARIA study cohort [4], consecutively recruited from patients followed in nine different cardiological centers in the Veneto Region in the northeast of Italy. 

Inclusion criteria were: aged at least 18 years old, having been implanted with an Evia© or Estella© pacemaker, with a Lumax© ICD-CRT, or with an ICD (Biotronik, Berlin, Germany). The system had to be compatible with a remote monitoring system with daily data transmission, and the patient’s residence had to be within 20 km of the pollution monitoring station. Patients were recruited between 1 April 2011, and 30 September 2012, with a minimum follow-up of 1 year. Then, the patients were scheduled to have a closing visit within 1 subsequent year.

As in a previously published work on the same study cohort [4], patients were excluded if they had clinically manifested heart failure, concomitant illness or a severe disorder that severely limited life expectancy; any medical or surgical disorder that, at the discretion of the investigator, placed the patient at high risk for adverse clinical events for participation in the study; a previous 2-year history of malignancy of any organ system, regardless of whether it was treated, including leukemia and lymphoma (with the exception of basal cell carcinoma of the skin), if there was evidence of local recurrence of metastasis; a history of drug or alcohol abuse in the last 2 years.

All patients who complied with these criteria were included. A first analysis focused only on patients with fewer than 6 AF episodes in a year to exclude patients with frequent AF. Patients with a higher number of AF events per year were included in a secondary analysis. This study is registered with ClinicalTrials.gov, number NCT01723761. 

### 2.2. Procedures

Diagnostic data recorded by the device during the day were transmitted daily to a mobile transmitter placed in the patient’s house. This transmitter sent data in turn through a Global System for Mobile Communications network to the Biotronik Home Monitoring Service Center where the data could be examined by physicians. 

Biotronik detected AF in its devices as “Atrial Burden” (AB), i.e., the percentage of time a patient was in AF during the day. An AB detected by the device, in which the time a patient was in AF throughout one day was ≥0.5%, was considered as an AF event.

### 2.3. Environmental Data

Daily PM_2.5_ and PM_10_ concentrations were obtained from permanent monitoring stations of the Regional Agency for Environmental Prevention and Protection of the Veneto Region (ARPAV), located throughout the Veneto region of Italy. In addition, median daily values of temperature, humidity, and pressure were collected from the meteorological center of the same agency, and they were considered as possible confounders.

Patients were associated with exposure data from the monitoring station that was nearest to their residence.

### 2.4. Statistical Analyses

Continuous variables are expressed as median (I and III quartiles), whereas categorical variables are represented with frequencies (percentages). In the present study, AB equal to 0 was labeled as an absence of AB, whereas AB greater than 0 was the presence of AB. 

A multivariable logistic model was implemented to assess the effects of the daily level of PM_2.5_ and PM_10_ on the risk of AB. Models were adjusted for three groups of variables: (i) environmental variables, i.e., the median daily levels of humidity, pressure, and temperature; (ii) clinical variables, i.e., β-blocker therapy and antiarrhythmics drugs; (iii) demographical variables, i.e., age and sex. The association between pollutants and AB was flexibly modeled with a restricted cubic spline function to allow for potential nonlinear relationships. A robust sandwich variance estimator was used to account for repeated measurements of the same subjects during follow-up [21]. Firth’s bias reduction method, which produces finite parameter estimates by means of a penalized maximum likelihood estimation, was used to reduce the bias introduced in the coefficients’ estimates of the logistic model by the small number of events [22,23]. The association was evaluated first in patients with fewer than 6 events per year. 

Different sets of patients were considered in a second analysis to evaluate how the estimated association was sensitive to the AB episodes inclusion criterion. The models were fitted on different samples of patients obtained by increasing the number of AB episodes per patient per year. The relationship between the number of AB episodes per patient per year and the association between AB and PM was then assessed.

As sensitivity analyses, we performed distributed lag models [24], including the lag from 0 to 2 of the pollutants, temperature, pressure, and humidity on the population with fewer than 6 AB episodes. 

Results are represented as Odds Ratio (OR) and relative 95% Confidence Interval (CI) for an increase in the pollutant concentration of 50 µg/m^3^ over the WHO threshold.

All of the analyses were implemented with R-System [25]. The *rms* [26] and *brglm* [27] libraries were used to fit the models.

## 3. Results

A total of 145 patients living in 137 municipalities of the Veneto region were included in the study cohort. The clinical features of the patients are presented in Table 1.

PM_2.5_ exceeded the World Health Organization (WHO) threshold of 25 μg/m^3^ for 26% of the days, whereas PM_10_ was detected above the threshold of 50 μg/m^3^ for 18% of the days during the follow-up (Table 2).

Overall, 26 episodes of AB were observed. The odds ratios (OR) of the models indicated a significant association between AB and daily levels of both PM_2.5_ and PM_10_ (Table 3).

Univariable logistic model for pollutants modeled with a restricted cubic spline; logistic model with Firth’s correction adjusted for antiarrhythmic and β-blockers therapy, age, sex, median daily pressure, temperature and humidity.

**Figure 1:**
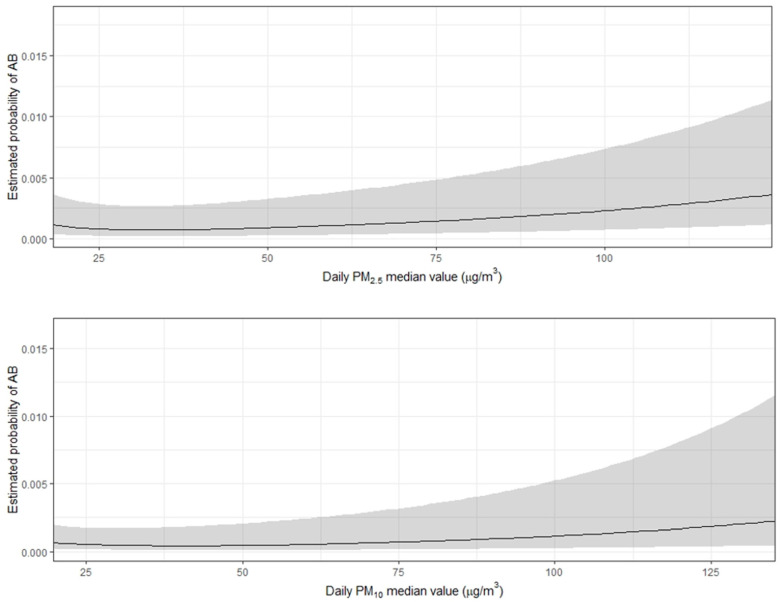
Plot of the estimated daily probability of atrial burden (AB) at different concentrations of particulate matters of less than 2.5 μm (PM_2.5_) and less than 10 μm (PM_10_) in aerodynamic diameter.

The risk of AB onset increases nonlinearly for concentrations above the WHO thresholds. PM_2.5_ and PM_10_ seem to increase the AB risk, mostly at higher concentrations (Figure 1).

**Figure 2:**
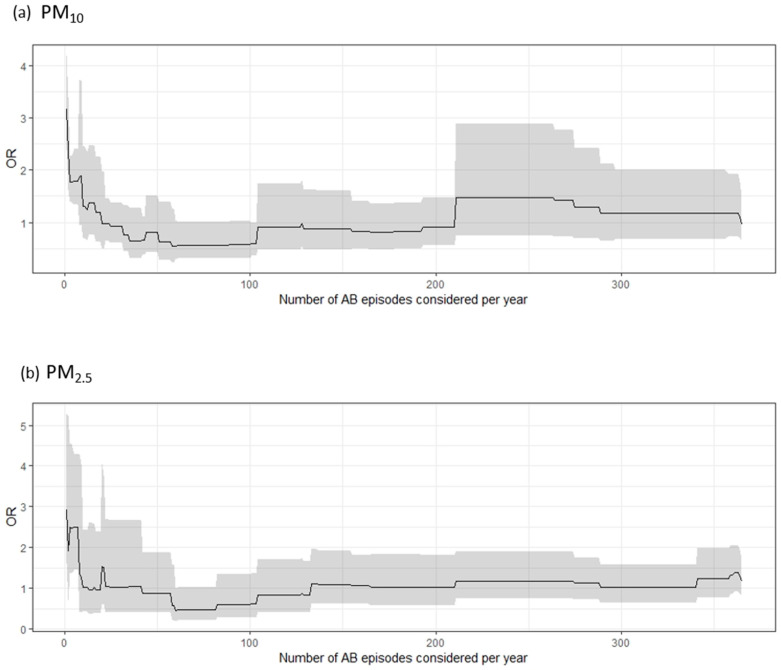
Increasing number of atrial burden (AB) episodes considered in the analysis and relative OR and confidence interval (in gray) for particulate matters of less than 10 μm (PM_10_) (**a**) and less than 2.5 μm (PM_2.5_) (**b**) in aerodynamic diameter.

In a second analysis, the risk of AB in association with PM was evaluated by consecutively increasing the number of AB episodes per year considered, with the consequent increase in population size (Figure 2). On the *x*-axis, the number of AB episodes per year is reported. Each point on the *x*-axis corresponds to different sets of subjects with a given number of AB episodes in a year. On the *y*-axis, the ORs with relative 95% CIs for a given set of subjects are depicted.

Including up to 365 AB episodes in a year, the total number of patients considered for the analysis was 201 and the total number of AB episodes recorded was 13,760. The value that identifies a statistically significant association between PM and the risk of AB was found to be eight AB episodes during a year. Therefore, if a patient experiences fewer than eight episodes, the association is positive and statistically significant for both PM_2.5_ and PM_10_, whereas if the number of episodes is greater to or equal to eight, air pollution seems not to affect the risk of AB (Figure 2).

Sensitivity analyses showed that the significant effect of PM_10_ and PM_2.5_ on AB risk is present at lag 0, whereas no significant effect is shown for air pollution at lags from 1 to 2 (Table 4).

## 4. Discussion

In this multicentric longitudinal study of patients implanted with an ICD, an ICD-CRT, or a pacemaker, an association was found between AB and daily levels of both PM_2.5_ and PM_10_. By recruiting patients with these types of devices, it was possible to also capture asymptomatic AF.

Our results are in line with those of other epidemiological and observational investigations that found an association between PM exposure and AF. In fact, it has been observed there is a significant association between AB and daily levels of both PM_2.5_ and PM_10._ The first evidence of the role of air pollution in triggering AF has been highlighted by Liao and colleagues [17]. Acute exposure to PM_2.5_ was indeed found to be associated with predictors of AF with more complex P-wave morphology and PR duration in a sample of subjects without severe cardiac problems [17].

A recent study conducted in China on patients who had cardiac implantable electronic devices showed an increased risk of AF onset in association with high levels of PM_2.5_ and PM_10_ [16]. Moreover, two studies found an association between air pollution and cardiac arrhythmias’ hospitalizations [10,28], and both reported that the average air pollution concentrations recorded in the study period exceeded the WHO thresholds. Conversely, the study conducted by Rich and colleagues in a less polluted area observed a positive but nonsignificant increased risk between paroxysmal atrial fibrillation and PM_2.5_ in ICD implanted patients [20]. Link and colleagues found a significant association between AF and increased levels of PM_2.5_ in the 2 h prior to the AF onset, only looking at the highest quartile of PM values distribution [13], thus supporting the hypothesis that PM only increases the risk of arrhythmias at medium to high PM concentrations.

Nevertheless, some studies focused on AF hospitalizations did not find an association between increased PM concentration and the risk of hospitalization [11,12,19]. In these studies, the outcome definition did not include people with asymptomatic AF, and the target population was the general population, not exclusively high-risk subjects. Indeed, patients implanted with ICDs, ICD-CRT, or pacemakers could be defined as high-risk, as they might be more susceptible to air pollution due to their underlying pathological condition [19].

The primary analyses conducted in this paper were focused on patients with a maximum of six AB episodes in a year in order to exclude patients in which a frequent AB onset could be mostly related to electric remodeling phenomena and not to air pollution. Therefore, in subjects with persistent or permanent AB, the effects of air pollution on AB onset could be underestimated.

To assess this hypothesis, the role of air pollution on the risk of AB occurrence was evaluated in an increasing number of patients by also considering the ones first excluded because of their high number of AB episodes in a year. In this way, the analysis was repeated 365 times, starting from patients who had only one AB episode per year and including progressively more patients until finally considering patients with 365 events per year. The results showed that for both PM_2.5_ and PM_10_ there was a significant positive effect on AB occurrence from one episode to seven episodes considered per year.

This result is in accordance with what was already reported in the literature [13], where PM seems to play a role only in patients with infrequent AF episodes. Furthermore, Link and colleagues found a statistically significant association between PM_2.5_ and AF onset in ICD-implanted patients with fewer than 20 episodes, and when looking at the whole population, the association was still positive but nonsignificant [13]. 

Mechanisms through which PM could lead to AF have not been completely understood so far. One major hypothesis is that inhaled PM activates sensory receptors in the lungs, leading to alterations in the autonomic tone that could result in an increased risk of arrhythmias [29,30]. Furthermore, it has been hypothesized that PM triggers inflammation and oxidative stress [31]. In particular, exposure to air pollution seems to increase the levels of C-reactive protein [32] that are, in turn, associated with an increased risk of developing AF [33,34].

The wide range of results reported in these years may be due to the different concentration levels of ambient PM, different components of PM, population susceptibility, and number of AF episodes per year considered, but a common theme is now emerging.

### Study Limitations

This study has some limitations. First, aiming to evaluate the effects of PM in patients who have infrequent AB episodes meant that the patient sample was limited. Moreover, the low number of AB episodes per patient per year considered in the first analysis led to a low number of total AB episodes observed. However, this problem was addressed using a logistic regression model with Firth’s correction, which reduced the bias in the maximum likelihood estimates of coefficients and consequently led to a more accurate stratification of the risk. Additionally, this statistical approach minimized the potential impact on estimates that may occur in such situations by specifically addressing the inflation of observations not associated with an event of interest. As persistent AB is mainly due to underlying pathologies, the real effect of air pollution on this subgroup cannot be estimated.

Moreover, no interactions between patients’ therapies were tested, and other possible confounding variables such as pulmonary disease, physical activity, vulnerability status or exposure to other pollutants have not been included.

The possible exposure misclassification due to the use of fixed monitoring stations that do not represent the real-time exposure of patients was partially controlled by the homogeneous distribution of PM in the Veneto region because of its morphological conditions. Moreover, the age of the patients suggests that most of them were not still working, so their movements due to work were limited. As such, the theoretical exposure was assumed to be at least 8 h.

## 5. Conclusions

In the current study, an association was found between AF and moderate air pollution exposure to both PM_2.5_ and PM_10_ in patients implanted with ICDs, ICD-CRT, and pacemakers with few episodes of AF. This finding is in accordance with studies indicating that there was a positive and significant association with high-moderate levels of pollutants.

## Figures and Tables

**Table 1:** Baseline patient characteristics.

		Patients (*n* = 145)
Men		109 (75.2)
Age (years)		72.00 [64.00, 77.00]
Smokers		12 (8.3)
Hypertensive cardiomyopathy		16 (11.0)
Myocardial infarction		40 (27.6)
Valvular heart disease		6 (4.1)
Dilated cardiomyopathy		45 (31)
Hypertrophic cardiomyopathy		2 (1.4)
**Treatments**		
Antiarrhythmics drugs		29 (20.0)
β-blockers		81 (55.9)
ACE inhibitors		68 (46.9)
Diuretics		74 (51.0)
Spironolactone		8 (5.5)
Calcium-antagonists		21 (14.5)
α-blockers		3 (2.1)
Ivabradine		1 (0.7)
Digitalis		5 (3.4)
Nitrates		7 (4.8)
Antiplatelet		73 (50.3)
Oral anticoagulant		31 (21.4)
NYHA class	I	45 (51.1)
	II	35 (39.8)
	III	8 (9.1)
	IV	0
**Comorbidities**		
Hypertension		70 (48.3)
Diabetes		30 (20.7)
Kidney failure		8 (5.5)
Previous stroke or TIA		11 (7.6)
Chronic Obstructive Pulmonary Disease		12 (8.3)

Data are *n* (%) or median [IQR]. NYHA = New York Heart Association. ACE = Angiotensin-Converting Enzyme. TIA = Transient Ischemic Attack.

**Table 2:** Pollutants data.

Pollutants	
PM_2.5_ (μg/m^3^)	16.00 [10.00, 26.00]
Days PM_2.5_ over the threshold	9697 (26.2%)
PM_10_ (μg/m^3^)	26.00 [15.50, 42.50]
Days PM_10_ over the threshold	3704 (18.3%)

Data are median [IQR] or *n* (%). PM_2.5_ and PM_10_ are particulate matter of less than 2.5 μm and 10 μm in aerodynamic diameter, respectively.

**Table 3:** Estimated effects of pollutants on atrial burden.

	Effects	Odds Ratio	95% CI	*p*-Value (Nonlinearity)
**Univariable model**				
PM_2.5_	75 vs. 25	1.98	1.22–3.22	0.002
PM_10_	100 vs. 50	2.64	1.02–6.83	0.249
**Adjusted model and Firth corrected**				
PM_2.5_	75 vs. 25	1.80	1.34–2.40	0.01
PM_10_	100 vs. 50	2.48	1.44–4.28	0.01

PM_2.5_ and PM_10_ are particulate matter of less than 2.5 μm and 10 μm in aerodynamic diameter, respectively.

**Table 4:** Firth’s logistic regression with delayed effects in time analyzed with a distributed lag model.

Pollutant	Effect	OR (95% CI) Lag 0	OR (95% CI) Lag 1	OR (95% CI) Lag 2
PM_2.5_	75 vs. 25	3.46 (1.36–8.74)	1.42 (0.56–3.59)	1.21 (0.70–2.10)
PM_10_	100 vs. 50	3.74 (2.52–5.55)	1.48 (0.90–2.42)	0.69 (0.44–1.06)

## Abbreviations

AB = atrial burden
AF = atrial fibrillation
CI = confidence interval
ICD = implantable cardioverter-defibrillator
ICD-CRT = cardiac resynchronization therapy defibrillators
IQR = interquartile range
OR = Odds Ratio
PM2.5 = particulate matter of less than 2.5 μm in aerodynamic diameter
PM10 = particulate matter of less than 10 μm in aerodynamic diameter
WHO = World Health Organization
